# Osteoblastic Solitary Plasmacytoma of Bone

**DOI:** 10.4274/tjh.galenos.2019.0218.0419

**Published:** 2019-05-03

**Authors:** Chrissa Sioka, Konstantinos Sakelariou, Alexandra Papoudou-Bai, Christos Tolis, Jihand Al-Boucharali, Andreas Fotopoulos

**Affiliations:** 1 School of Health Sciences, University Hospital of Ioannina Faculty of Medicine, Department of Nuclear Medicine, Ioannina, Greece; 2 School of Health Sciences, University Hospital of Ioannina Faculty of Medicine, Department of Pathology, Ioannina, Greece; 3 Oncoderm Center, Ioannina, Greece

**Keywords:** Plasmacytoma, Bone scintigraphy, Multiple myeloma

A 54-year-old woman was subjected to a routine annual chest X-ray for work license renewal, which showed a hyperdense lesion of the left 8th rib (Figure 1). A chest computed tomography (CT) scan documented this abnormality, which was considered to represent Paget’s disease, bone metastasis, or a primary bone tumor.

A whole-body bone scan showed increased radionuclide uptake (Figure 2), indicating an osteoblastic lesion in a large portion of the rib (arrows), with intense focal uptake (arrowhead).

Diagnostic biopsy and histological examination of a tissue specimen from the affected rib (Figure 3) revealed dense infiltration of plasma cells (Figure 3A, hematoxylin and eosin stain, 600^x^). Immunohistochemically, the cells expressed CD138 (Figure 3B, DAB, 200^x^) and CD38 (Figure 3C, DAB, 200^x^) and were IgA-positive (Figure 3D, DAB, 200^x^). Immunostaining showed lambda light-chain restriction (Figure 3E, DAB, 200^x^) with no expression of kappa light-chain (Figure 3F, DAB, 200^x^), consistent with plasma cell neoplasm. The bone marrow biopsy obtained from the left iliac crest was free of neoplastic invasion. An X-ray of the axial skeleton and long bones and a CT scan of the skull and thorax were performed, which did not reveal any additional bone lesions. Laboratory test results demonstrated normal creatinine (0.73 mg/dL) and total calcium (9.6 mg/dL) levels. The results of the complete blood count showed a white blood cell count of 3.39x10^3^/µL with no other remarkable findings. B2 microglobulin was 2091 µL (normal range: 700-3400) and alkaline phosphatase was 40 IU/L (normal range: 30-125). Serum free light-chains were absent and there was no serum or urine monoclonal paraprotein detection. Taking into consideration all of the above-mentioned findings, a diagnosis of osteoblastic solitary plasmacytoma was made.

Solitary osseous plasmacytoma consists of a mass of neoplastic monoclonal plasma cells associated with bone osteolysis [1,2]. During diagnostic workup, fludeoxyglucose-positron emission tomography should be performed, if available, to rule out smoldering multiple myeloma and monitor response to treatment [3,4]. Solitary osteolytic bone plasmacytomas, although rare, have been reported in several bone areas such as the lumbar spine vertebra, the sternum, or even the ribs [2,5]. However, plasmacytoma exhibiting osteoblastic characteristics such as in our case is extremely rare and deserves further investigation.

## Figures and Tables

**Figure 1:**
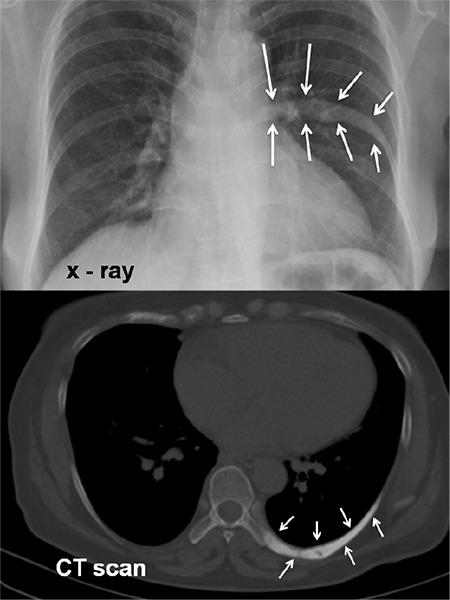
Chest X-ray (upper panel) revealing a hyperdense lesion in the left 8^th^ rib (arrows); the computed tomography scan of the chest (lower panel, arrows) documented the abnormality. CT: Computed tomography.

**Figure 2:**
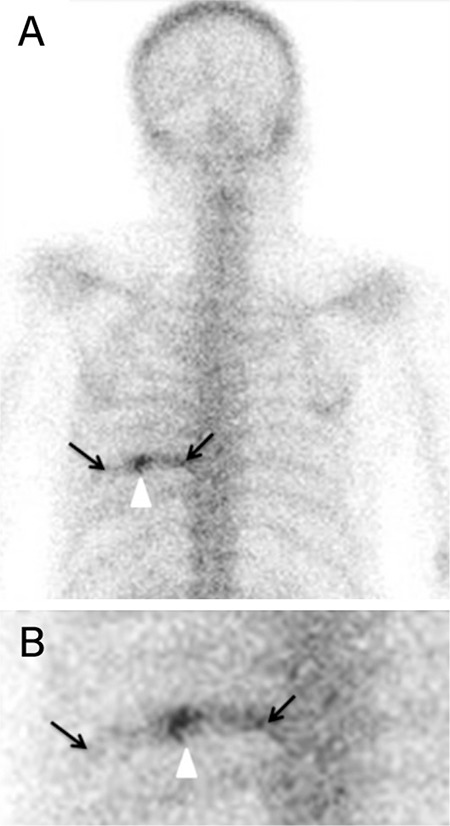
Whole-body bone scan with Tc-99m-methylene diphosphonate demonstrated increased radionuclide uptake, indicating an osteoblastic lesion in a large portion of the rib (arrows), with intense focal uptake (arrowhead).

**Figure 3:**
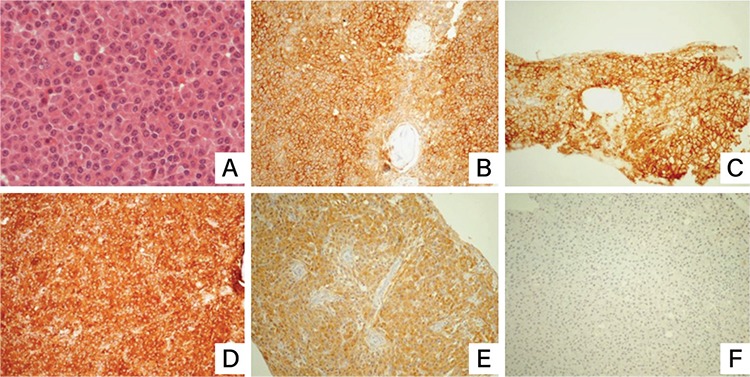
Histological examination of the bone lesion revealed plasma cell infiltrate (3A, hematoxylin and eosin staining, magnification 600^x^). The neoplastic cells were CD138-positive (3B, DAB, magnification 200^x^) and CD38-positive (3C, DAB, magnification 200^x^) and expressed IgA (3D, DAB, magnification 200^x^). Immunostainings for kappa and lambda light chains showed cytoplasmic light chain positivity (3E, DAB, magnification 200^x^) and absence of kappa light chain (3F, DAB, magnification 200^x^).
